# Effect of Three Commercially Available Extenders Containing Phospholipids of Different Sources on Skopelos Buck Liquid-Stored Sperm Quality

**DOI:** 10.3390/vetsci11100494

**Published:** 2024-10-11

**Authors:** Aikaterini Saratsi, Foteini Samartzi, Dimitrios Tsiokos, Ekaterini K. Theodosiadou, Ioannis Panagiotidis, Christina Ligda, Constantinos A. Rekkas

**Affiliations:** 1 Veterinary Research Institute, Hellenic Agricultural Organization-DIMITRA, ELGO Campus, Thermi, 57001 Thessaloniki, Greece; ksaratsi@vri.gr (A.S.); samartzi@vri.gr (F.S.); chligda@elgo.gr (C.L.); 2 Research Institute of Animal Science, Hellenic Agricultural Organization-DIMITRA, Paralimni, 58100 Giannitsa, Greece; tsiokosd@elgo.gr; 3 Laboratory of Physiology, Faculty of Veterinary Science, University of Thessaly, Trikalon 224, 43100 Karditsa, Greece; etheodosiadou@uth.gr; 4 Department of Animal Reproduction & Artificial Insemination, Directorate of Veterinary Center of Thessaloniki, Ministry of Rural Development and Food, 9 Verias Str., 57008 Thessaloniki, Greece; giannis.panagiotidis@gmail.com

**Keywords:** buck semen, liquid storage, extender, soy lecithin, egg yolk, plant phospholipids

## Abstract

**Simple Summary:**

This study aimed to compare the effect of three commercially available extenders based on phospholipids [soy lecithin (SL, OviXcell^®^), plant phospholipids (PP, AndroMed^®^) or egg yolk lecithin (EY, Steridyl^®^)] and a basic Tris-citrate-glucose home-made extender (no phospholipids), on liquid-stored semen quality, at 5 °C for a 48 h storage period. Overall, the three supplemented extenders enhanced liquid-stored spermatozoa viability, acrosome and membrane integrity, in comparison to the basic extender. SL and EY extenders preserved viability more effectively than the PP extender, while total motility was higher in the PP extender, compared to the SL extender. It is worth noting that EY Steridyl^®^ was more effective in maintaining a combination of core sperm qualities, such as spermatozoa motility and acrosome integrity. Fertility trials are necessary to clarify these effects.

**Abstract:**

The effect of four extenders on buck semen quality parameters was examined during a 48 h liquid storage. Semen was collected from six Skopelos bucks and diluted in the following extenders, containing: soy lecithin (SL, OviXcell^®^), plant phospholipids (PP, AndroMed^®^), egg yolk lecithin (EY, Steridyl^®^), or no phospholipids (basic extender). Samples were stored at 5 °C for 48 h and assessed at 0, 24 and 48 h for viability (eosin-nigrosin), acrosome integrity (SpermBlue^®^), membrane functional integrity (HOST), mitochondrial function (Rhodamine 123/SYBR-14/PI) and motility parameters (CASA). No significant reduction in total or progressive spermatozoa motility and mitochondrial function was observed at 24 h, whereas they all dropped significantly at 48 h, in all extenders. Spermatozoa viability, cell membrane functionality and acrosome integrity dropped progressively (0 h > 24 h > 48 h) in all groups. No significant difference among extenders was observed concerning spermatozoa mitochondrial function. Overall, spermatozoa viability, cell membrane functionality and acrosome integrity were higher in the three commercial extenders, compared to the basic extender. SL and EY extenders (OviXcell^®^ and Steridyl^®^, respectively) preserved viability more effectively than the PP extender (AndroMed^®^). Total motility was higher in the PP extender, compared to the SL extender. Spermatozoa acrosome integrity tended to be higher in the EY extender compared to all the other extenders. Further investigation of the protective potential of different types of cryoprotectants on liquid buck semen storage is important.

## 1. Introduction

Efficient herd management and genetic improvement are prerequisites for competent goat production. Artificial insemination (AI) is the basis of genetic improvement programs [1]. Semen liquid storage or cryopreservation promote the dispersion of valuable genetic material among goat farms [2]. Cryopreserved semen is valuable in gene banking and long-term storage of doses of elite males. However, the cost of a frozen dose is higher than the cost of a liquid-stored dose [3]. Moreover, liquid-stored semen grants easier transportation and handling than frozen semen [4], while it yields higher fertility rates in inseminated goats, since its performance is not affected as much by cervical or intracervical deposition [5]. Insemination with liquid-stored buck semen is currently commonly performed in goats, mostly within 24 h after collection. The time period that liquid-stored buck semen maintains its quality characteristics and fertilizing ability is of paramount importance for the success of artificial insemination in the framework of breeding programs [6,7].

An extender or diluent is a liquid medium utilized for the extension and protection of spermatozoa, during storage and transportation, until used for AI. An effective extender sustains osmotic pressure and pH of diluted semen (salts and buffers), supports spermatozoa metabolism (sugars), prevents bacterial growth (antibiotics), cold shock and cryodamage (cryoprotectants). Two categories of cryoprotectants are used, according to their ability to enter the spermatozoa cell membrane: permeating (e.g., glycerol) and non- permeating (e.g., egg yolk, skimmed milk, soy lecithin) [8,9,10].

Glycerol is the most commonly used permeating cryoprotectant, since it prevents intracellular crystallization [8]. The addition of 5% glycerol preserves sperm motility and kinetics in frozen-thawed goat semen, while it also protects spermatozoa normal morphology [11].

Depending on the origin of the non-permeating cryoprotective agent, semen extenders currently used in semen cryopreservation and liquid storage protocols fall into two groups: egg yolk-based [12,13] or plant phospholipid-based extenders [14,15]. Egg yolk is the main cryoprotectant used for semen cryopreservation, as it protects the cell and acrosome membrane of spermatozoa from both cold shock and cryodamage [16], while it preserves spermatozoa motility and mitochondrial function [17]. When buck semen is extended in egg yolk-based cryoprotectants, seminal plasma is removed, through centrifugation, to avoid the harmful effects of the interaction of buck seminal plasma with egg yolk [18]. However, the removal itself has detrimental effects on frozen-thawed buck semen [19,20,21]. In liquid storage in egg yolk extenders, removal of buck seminal plasma is not always considered necessary, as fertility trials have proven [22,23,24].

Plant phospholipid sources, and especially soy lecithin, have been used as substitutes for cryoprotectants of animal origin, for the past 15 years [25]. Soy lecithin differs in lipid composition, in comparison to egg yolk [26,27] and has been tested, as an alternative for cryoprotectants of animal origin, in buck semen cryopreservation, both in in vitro experimental models and in fertility trials, with varying and sometimes contradicting results and conclusions [21,28,29,30,31].

The design of extenders, concerning cryoprotectants, is targeting the amelioration of cryopreservation protocols. An important part of the action of cryoprotectants though, is aiming at preventing the first shock of cooling until 5 °C, during the semen equilibration period, commonly known as cold shock [32,33]. Cold shock is described as damage to the integrity and permeability of the spermatozoa cell membrane, modification of intracellular enzyme activity, lipid transformation and ion redistribution, finally causing acrosomal and mitochondrial membrane abnormalities, which result in impaired motility [34]. Liquid-stored semen undergoes cold shock and therefore cryoprotectants are important to its quality and fertilizing ability [35].

The protective effects of both egg yolk- and plant phospholipid-based extenders on buck spermatozoa, diluted in different commercial extenders, depend upon the structure, function and concentration of cryoprotectants in each extender [25,36].

Breed differences might also affect the quality and cryotolerance of buck semen [37], since they strongly affect ejaculate concentration and volume, percentage of abnormal spermatozoa and sperm quality characteristics [38]. Significant variations in cryotolerance have been reported, not only among individual bucks within a breed, but also among breeds [39,40]. The cryotolerance of Skopelos buck semen has not yet been adequately studied and described.

The present study was conducted in order to provide some first insight into the sensitivity of buck semen to different cryoprotectants, included in standard commercial extenders, during a 48 h liquid storage. Viability, cell and acrosome membrane integrity, mitochondrial membrane potential and motility parameters were recorded to compare the effect of semen extenders on sperm quality.

## 2. Materials and Methods

### 2.1. Selection of Bucks, Semen Collection and Handling

This study was performed at the facilities of the Department of Animal Reproduction & Artificial Insemination, Directorate of Veterinary Center of Thessaloniki, Ministry of Rural Development and Food. All animal procedures were performed in accordance with the European Union Regulation 2010/63.

Skopelos bucks were selected on the basis of data collected from the genetic improvement program of the breed, as performed by the Breeders’ Association (Livestock Cooperative of Volos—Skopelos goat breed). The Association maintained a comprehensive database, that encompassed genealogical and production records of the breeding population. By examining the genealogical information, non-related animals were selected and production data from their relatives were used to objectively evaluate the potential of each candidate buck.

Six Skopelos bucks, 2–3 years old, were housed in individual pens, under natural daylight conditions (40″ 68′ of North Latitude). They were fed mixed grass hay and concentrate, as needed to maintain a healthy body condition score, and had access to fresh water, ad libitum.

Semen was collected, with an artificial vagina, twice in a month (June, non-breeding season). A maximum of two ejaculates, with a minimum of 15 min interval between ejaculations, were collected, per animal, per collection date. Semen samples were placed in a water bath at 37 °C, immediately after collection. Ejaculates from the same buck were at this stage pooled. Initially, semen volume was recorded and spermatozoa concentration was evaluated by a photometer (DR Lange LP 1 Photometer, Minitüb GmbH, Germany). Spermatozoa motility was subjectively evaluated, always by the same person, using a standard dilution (1:200) in PBS (37 °C), under a phase-contrast microscope (×400). At least five random fields were observed for each sample. Extended samples were kept at 4 °C for 1 h, before sperm quality parameters were evaluated (0 h). Eosin-nigrosin stain was used for the evaluation of spermatozoa viability and morphology (200 spermatozoa per slide were examined, always by the same person). The ejaculates that provided >70% live and motile spermatozoa and <5% morphological abnormalities were further processed [41]. Every ejaculate was split into four parts and each part was diluted in one of the four extenders to the final concentration of 400 × 10^6^ spermatozoa/mL; seminal plasma was not removed.

### 2.2. Semen Extenders and Dilution

Three commercially available extenders containing phospholipids [soy lecithin-based (SL, OviXcell^®^; IMV Technologies, Saint Uuen sur Iton, L’Aigle, France); plant phospholipid-based (PP, AndroMed^®^; Minitüb GmbH, Germany); sterilized egg yolk lecithin-based (EY, Steridyl^®^; Minitüb GmbH, Tiefenbach, Germany)] and a basic Tris-citrate-glucose home-made extender (no phospholipids), were used. All four extenders contained salts, electrolytes and buffers, diluted in ultra-pure water. All of the extenders contained sugars and antibiotics. Only the three commercial extenders contained glycerol and non- permeating cryoprotectants. The basic extender [glucose monohydrate 37 g/L, EDTA-Na 1.25 g/L, Na-Citrate 5.0 g/L, KCl 0.75 g/L, Mops 15 g/L, Bes 1.0 g/L, glycin 1.0 g/L)], was prepared in our Lab and the pH was regulated to 7.0 with Tris (crystallic form). Gentamycin served as an antimicrobial agent (500 μg/mL). Semen evaluation was performed at 0, 24 and 48 h of liquid storage.

### 2.3. Semen Evaluation

#### 2.3.1. Viability

Spermatozoa viability was assessed using the eosin-nigrosin stain (1% eosin, 3% nigrosin, 3% sodium citrate, 100 mL distilled water). On each slide, 15 μL of semen and an equal amount of eosin-nigrosin stain were mixed and smeared on the slide with a coverslip. Slides were left to airdry and were evaluated for spermatozoa viability. At least 200 spermatozoa per slide were counted (×400), always by the same person.

#### 2.3.2. Cell Membrane Functional Integrity

Assessment of spermatozoa cell membrane functional integrity was performed by the hypo-osmotic swelling test (HOST), according to Fonseca et al. [42]. At least 200 spermatozoa per slide were counted (×400), always by the same person.

#### 2.3.3. Acrosome Integrity

Acrosome integrity was evaluated according to [43], using the SpermBlue^®^ stain kit (Microptic, Automatic Diagnostic Systems, Barcelona, Spain). At least 200 spermatozoa per slide were counted (×400), using a blue filter (Leica DMLB), always by the same person.

#### 2.3.4. Mitochondrial Membrane Function Combined with Viability

Mitochondrial function was evaluated with Rhodamine 123 [44], while a concurrent viability assessment was performed by a SYBR-14/PI assay [44,45]. Samples were exposed to the three dyes in the dark, at room temperature (20–25 °C). By the end of a 25 min incubation period, samples were assessed (×400), under a fluorescent microscope (Leica DM 2000, Leica Microsystems, Ltd., Balgach, Switzerland), always by the same person.

#### 2.3.5. CASA Motility and Kinetics

Spermatozoa motility parameters were evaluated by a Computer Assisted Spermatozoa Analysis (CASA) system (Sperm Class Analyser^®^, Microptic S.L., Automatic Diagnostic Systems, Spain) and a microscope (AXIO Scope A1, Zeiss, Germany), equipped with a heating stage and a camera (Basler scA780 54fc, Germany). The analysis was performed by Sperm Class Analyser^®^ software (SCA^®^ v.6.3.; Microptic S.L., Automatic Diagnostic Systems, Spain). The CASA configurations were as follows: (1) at least 5 fields recorded (×100) for each semen sample, (2) >500 spermatozoa, (3) 25 frames/sec, (4) region of particle control 3–70 microns, (5) progressive movement of >80% of the straightness parameter.

For each sample, 10 μL of semen was placed on the preheated (37 °C) Makler chamber (Makler^®^ counting chamber, 10 μm deep, Sefi Medical Instruments, Israel) and the following CASA motility parameters were evaluated: (1) total motility (%), (2) progressive motility (%) and (3) kinetic parameters [curvilinear velocity (VCL, µm/s), straight-line velocity (VSL, µm/s), average path velocity (VAP, µm/s), linearity (LIN, %), straightness (STR, %), oscillation (WOB, %), amplitude of lateral head displacement (ALH, μm) or beat/cross frequency (BCF, Hz].

### 2.4. Statistical Analysis

All data obtained from this study were analyzed using SPSS (SPSS 20.0 for Windows; SPSS, Chicago, IL, USA). Univariate analysis of variance (univariate ANOVA) was applied followed by Duncan’s multiple range test; homogeneity of variances was evaluated with a Levene test and *p* < 0.05 was set as the minimum level of significance. Data are presented as mean ± standard error (SE).

## 3. Results

A total of 144 semen samples were evaluated for viability, cell membrane integrity, acrosome membrane integrity, mitochondrial functionality and motility.

### 3.1. Viability

**Figure 1:**
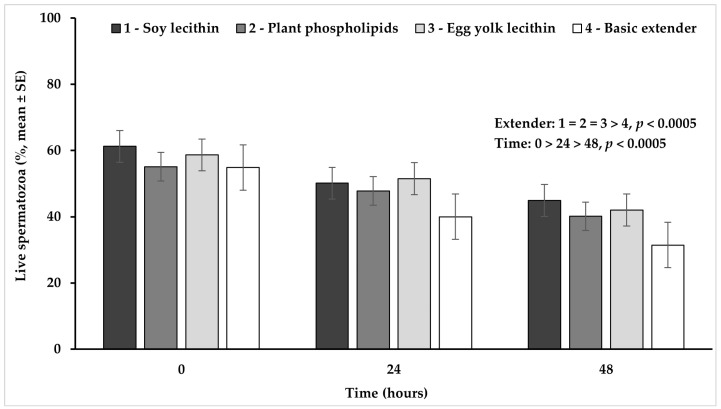
Percentage (mean ± SE) of live spermatozoa measured in Skopelos buck semen diluted with extenders containing soy lecithin (OviΧcell^®^), plant phospholipids (AndroMed^®^), egg yolk lecithin (Steridyl^®^) or no phospholipids (basic extender) after 0, 24 and 48 h of liquid storage (*p* is provided for comparisons between extenders within time and between time within extenders).

The percentage of live spermatozoa was significantly higher (*p* < 0.02) in the extender based on soy lecithin or on egg yolk lecithin, while it tended to be higher (*p* = 0.088) in the extender based on plant phospholipids, compared to the basic extender (no phospholipids). No significant difference (*p* > 0.05) was observed among extenders containing phospholipids (Figure 1).

The percentage of live spermatozoa appeared significantly higher at hour 0 in comparison to hour 24 and it further declined significantly at hour 48 (*p* < 0.0005), in all experimental groups (Figure 1).

### 3.2. Cell Membrane Functional Integrity

**Figure 2:**
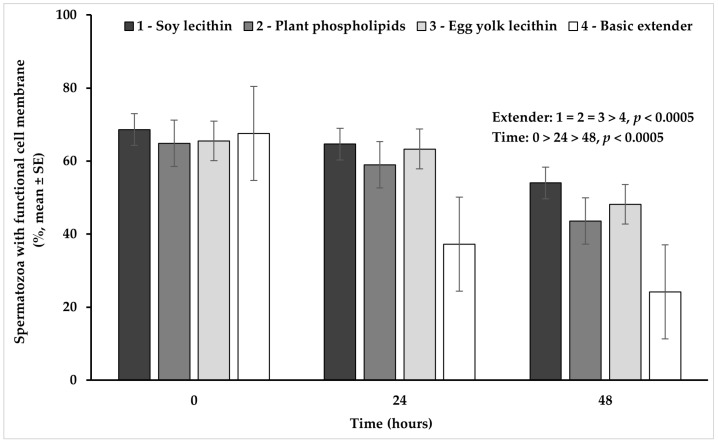
Percentage (mean ± SE) of spermatozoa with a functional cell membrane measured in Skopelos buck semen diluted with extenders containing soy lecithin (OviΧcell^®^), plant phospholipids (AndroMed^®^), egg yolk lecithin (Steridyl^®^) or no phospholipids (basic extender) after 0, 24 and 48 h of liquid storage *p* is provided for comparisons between extenders within time and between time within extenders).

Univariate analysis of variance revealed a significant (*p* < 0.05) interaction between extender (soy lecithin, plant phospholipids, egg yolk lecithin, basic extender; n = 48) and time (0, 24, 48 h; n = 36) (Figure 2).

In detail, spermatozoa with a functional cell membrane appeared in a significantly higher percentage in all extenders containing phospholipids, compared to the basic extender (*p* < 0.0005). No significant difference (*p* > 0.05) was observed among extenders containing phospholipids. Moreover, the percentage of spermatozoa with a functional cell membrane appeared significantly higher at hour 0, in comparison to hour 24 and it further declined significantly at hour 48 (*p* < 0.0005), in all experimental groups (Figure 2).

### 3.3. Acrosome Integrity

**Figure 3:**
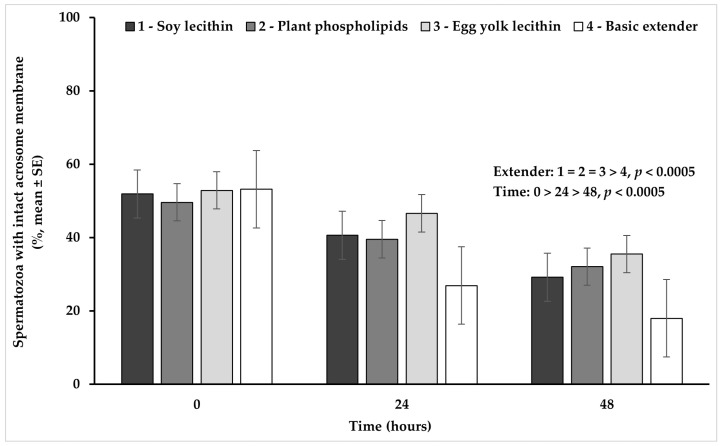
Percentage (mean ± SE) of spermatozoa with an intact acrosome membrane measured in Skopelos buck semen diluted with extenders containing soy lecithin (OviΧcell^®^), plant phospholipids (AndroMed^®^), egg yolk lecithin (Steridyl^®^) or no phospholipids (basic extender) after 0, 24 and 48 h of liquid storage (*p* is provided for comparisons between extenders within time and between time within extenders).

Univariate analysis of variance revealed a significant (*p* < 0.02) interaction between extender (soy lecithin, plant phospholipids, egg yolk lecithin, basic extender; n = 48) and time (0, 24, 48 h; n = 36) (Figure 3).

In detail, spermatozoa with an intact acrosome membrane appeared in a significantly higher percentage in all extenders containing phospholipids, compared to the basic extender (*p* < 0.0005). Furthermore, spermatozoa with an intact acrosome membrane tended to appear (*p* = 0.080) in a higher percentage in the egg yolk-based extender, than in the soy lecithin- or plant phospholipid-based extenders. The percentage of acrosome integrity appeared significantly higher at hour 0, in comparison to hour 24 and it further declined significantly at hour 48, in all experimental groups (*p* < 0.0005) (Figure 3).

### 3.4. Mitochondrial Membrane Function Combined with Viability

**Figure 4:**
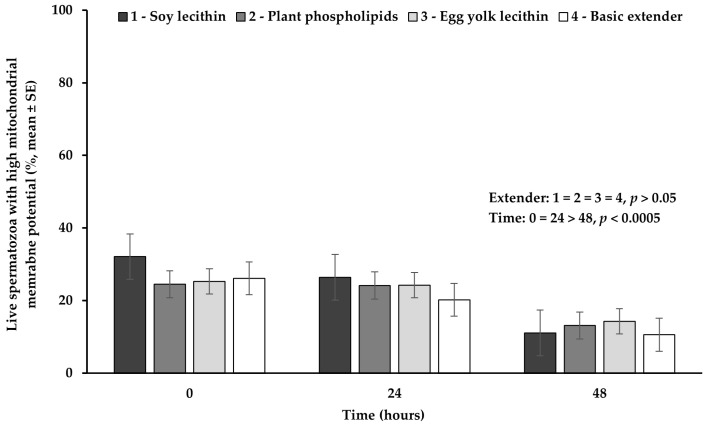
Percentage (mean ± SE) of live spermatozoa with high mitochondrial membrane potential measured in Skopelos buck semen diluted with extenders containing soy lecithin (OviΧcell^®^), plant phospholipids (AndroMed^®^), egg yolk lecithin (Steridyl^®^) or no phospholipids (basic extender) after 0, 24 and 48 h of liquid storage (*p* is provided for comparisons between extenders within time and between time within extenders).

The percentage of live spermatozoa with high mitochondrial membrane potential did not significantly differ (*p* > 0.05) among the four extenders studied (Figure 4).

The percentage of live spermatozoa with high mitochondrial membrane potential appeared significantly higher (*p* < 0.0005) at hour 0 or 24, compared to hour 48, while between 0 and 24 h no significant difference (*p* > 0.05) was observed (Figure 4).

### 3.5. CASA Motility and Kinetics

#### 3.5.1. Total Motility

**Figure 5:**
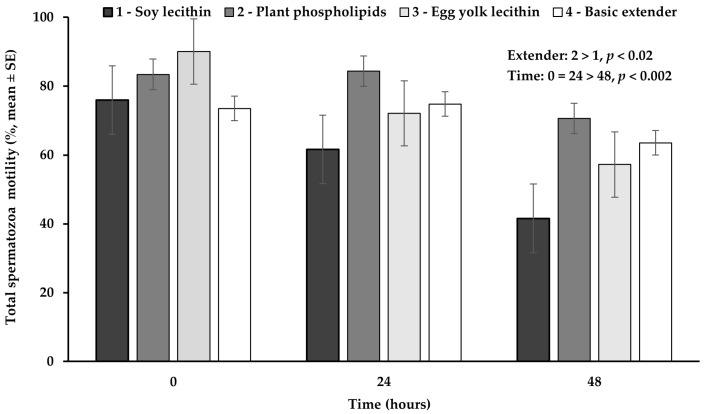
Percentage (mean ± SE) of total spermatozoa motility measured in Skopelos buck semen diluted with extenders containing soy lecithin (OviΧcell^®^), plant phospholipids (AndroMed^®^), egg yolk lecithin (Steridyl^®^) or no phospholipids (basic extender) after 0, 24 and 48 h of liquid storage (*p* is provided for comparisons between extenders within time and between time within extenders).

Total spermatozoa motility appeared significantly higher (*p* < 0.02) in the extender based on plant phospholipids, compared to the extender based on soy lecithin, while no significant difference (*p* > 0.05) was observed among all the other extenders (Figure 5).

The percentage of total spermatozoa motility appeared significantly higher (*p* < 0.002) at hour 0 or 24, compared to hour 48, while between 0 and 24 h no significant difference (*p* > 0.05) was observed (Figure 5).

#### 3.5.2. Progressive Motility

**Figure 6:**
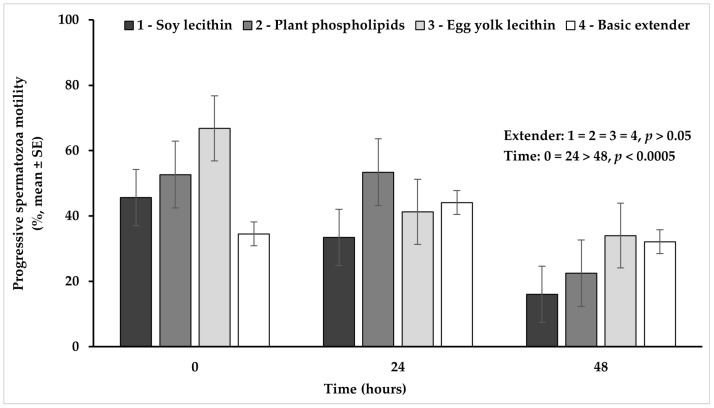
Percentage (mean ± SE) of spermatozoa progressive motility measured in Skopelos buck semen diluted with extenders containing soy lecithin (OviΧcell^®^), plant phospholipids (AndroMed^®^), egg yolk lecithin (Steridyl^®^) or no phospholipids (basic extender) after 0, 24 and 48 h of liquid storage (*p* is provided for comparisons between extenders within time and between time within extenders).

Progressive spermatozoa motility tended to be higher (*p* = 0.089) in the extender based on plant phospholipids or egg yolk lecithin, compared to the soy lecithin extender, while no significant difference (*p* > 0.05) was observed among all the other extenders (Figure 6).

The percentage of progressive spermatozoa motility appeared significantly higher (*p* < 0.0005) at hour 0 or 24, compared to hour 48, while between 0 and 24 h no significant difference (*p* > 0.05) was observed (Figure 6).

#### 3.5.3. Kinetic Parameters

VCL, VSL or VAP appeared significantly higher (*p* < 0.00005) in the extender based on egg yolk lecithin, compared to all other extenders. LIN or WOB appeared significantly higher (*p* < 0.00005) in the extender based on egg yolk lecithin or the basic extender, compared to all other extenders, while ALH appeared significantly higher (*p* < 0.00005) in the extender based on egg yolk lecithin or plant phospholipids, compared to all other extenders. BCF appeared significantly higher (*p* < 0.05) in the extender based on plant phospholipids compared to all other extenders, while no significant differences (*p* > 0.05) among extenders was found concerning STR (Table 1).

VCL, VSL, VAP, LIN, WOB, ALH and BCF showed reduction patterns similar to those of total and/or progressive motility with time, while no respective pattern regarding STR applied (*p* > 0.05) (Table 1).

## 4. Discussion

Most extenders, for both liquid-stored and cryopreserved buck semen, contain egg yolk and glycerol, as cryoprotectants. Egg yolk protects spermatozoa against cold shock and preserves motility, as semen is chilled from 37 °C to 4–5 °C [46]. Plant phospholipids are considered good alternatives to egg yolk, as has been proven in buck semen cryopreservation studies [31,47]. In the present study, sperm quality parameters were examined, to further understand the effect of the incorporation of phospholipids of different sources to semen extenders, on Skopelos buck semen quality.

As storage time increased (0, 24 and 48 h), sperm quality characteristics deteriorated gradually. Our results are in line with previous studies performed on buck semen liquid storage [22,35,48,49]. The same effect is reported for liquid-stored bull [50] or ram [51] semen. Low storage temperature reduces oxygen consumption and decelerates sperm metabolism. Yet, liquid-stored spermatozoa are under oxidative stress, which affects semen quality and fertilizing ability [52,53], often regardless of the extender type, dilution rate or storage conditions [51]. This is also reflected on the results obtained in the present study.

Concerning the effect of extender composition on spermatozoa motility, results from the present study indicate that spermatozoa total and progressive motility remained statistically unaffected for the first 24 h of storage. At 48 h, total and progressive motility dropped significantly. This is in accordance with other studies, which have shown that motility of liquid-stored buck semen is preserved for the first 24 h of storage [35,54]. However, it is in contrast to the results of studies on bull semen liquid storage, confirming that total and progressive motility decreases from the first 24 h of storage, regardless of extender composition [52,55]. In our study, progressive motility tended to be significantly higher in PP or EY, in comparison to SL. The abovementioned results from our study, concerning liquid-stored buck semen, are in contrast with the results of two studies on buck semen cryopreservation, that obtained higher post-thaw spermatozoa motility results from a soy lecithin-based extender (Bioxcell^®^), in comparison to egg yolk extenders [21,30]. Miguel-Jimenez et al. [56] found that the extender based on soy lecithin (Bioxcell^®^) causes an impairment of total and progressive motility of post-thaw bull spermatozoa, in comparison to a plant phospholipid-based extender (AndroMed^®^) or an egg yolk-based extender (Triladyl^®^). In line with our results is also a study on bull semen cryopreservation, which found no difference between a plant phospholipid-based extender (AndroMed^®^) and an egg yolk-based extender (Triladyl^®^) [57]. Interestingly, a study on bull semen that investigated the effect of four extenders on the post-thaw distribution of sperm subpopulations, based on motility patterns, reported that bull spermatozoa extended in a plant phospholipid-based extender (AndroMed^®^) had greater values of motion characteristics in their highly motile subpopulations, than those extended in a soy lecithin-based extender (Bioxcell^®^) [56]. These extra motile semen subpopulations are important for sperm longevity and fertilizing ability [58]. The Authors attribute the abovementioned differences to the composition of the extenders: in Bioxcell^®^, like in OviXcell^®^ in our case, plant phospholipids are contained in the form of lecithin, while AndroMed^®^ contains plant phospholipids of mixed origin, including other phospholipids than lecithin [56]. However, another study in bull semen cryopreservation reports an increase in post-thaw sperm motility when semen is diluted in AndroMed^®^ (PP), in comparison to an egg yolk extender [59], while there are a number of studies reporting a drop in post-thaw bull spermatozoa total and progressive motility when a soy lecithin-based extender is used as a substitute for egg yolk [50,60,61,62]. Papa et al. [63] observed the same result in frozen-thawed stallion spermatozoa. Finally, when different soy lecithin concentrations were tested in the liquid storage of ram semen, only the 2% lecithin extender yielded the same total motility results as the egg yolk extender [64], suggesting that the concentration of cryoprotectants plays a pivotal role in the outcome of these comparative studies.

Spermatozoa kinetic parameters generally followed the pattern of total and progressive motility concerning the effect of time of storage. They remained unchanged or slightly decreased during the first 24 h of storage and significantly deteriorated at 48 h. This is in accordance with other authors that report a gradual and concomitant decrease in sperm motility and kinetic parameters after the second day of liquid storage and as storage time increases [65,66]. Concerning the effect of the extender on sperm kinetic parameters, the egg yolk extender tended to be better at their preservation during storage, in this study. The extender composition did not influence STR at any sampling time. With the exception of BCF, all other kinetic parameters were higher in egg yolk, in comparison to the other extenders. BCF is the parameter most influenced by the high viscosity of the extender and it is reported to be higher in plant-based extenders, which are less viscous than the egg yolk extenders [67]. ALH appeared significantly higher in the egg yolk and the plant phospholipids extender, in comparison to the soy lecithin and the basic extenders. Our CASA results, both in motility and kinetic variables, are in contrast with the results of a similar study on buck semen cryopreservation [21], that reports that post-thaw progressive motility and kinetic parameters are preserved better in a plant phospholipid (Bioxcell^®^), than in a Τris-based egg yolk extender. Our results on total and progressive motility agree with a similar study on bull semen cryopreservation, but differ in the patterns the kinetic parameters have followed [61]. Celeghini et al. [61] have reported higher values of kinetic parameters in the plant phospholipid-based extender (Bioxcell^®^), in comparison to the egg yolk extender. These differences can be attributed to the different patterns of stress factors that are exercised upon the spermatozoa during liquid storage, in comparison to cryopreservation.

The percentage of live sperm with high mitochondrial membrane potential (MMP) followed the pattern of total and progressive motility. It had remained unchanged after 24 h of storage, while it dropped significantly after 48 h. This is in accordance with studies, which have proven that high MMP reflects the energy potential of spermatozoa and can be directly related to motility characteristics [68,69]. Interestingly, extender composition did not affect mitochondrial functionality, throughout the 48 h storage period. Similarly, in a buck semen cryopreservation study, that compared a soy lecithin and an egg yolk extender at preserving frozen-thawed spermatozoa viability and motility, the egg yolk extender granted superior protection to the abovementioned parameters, although these significant differences were not reflected on intracellular ATP concentrations [70].

The combination of cold shock with extended storage damages sperm membranes, since it causes irreversible changes in their phospholipid layers [71]. Cell membrane functionality is of pivotal importance during cold storage of spermatozoa and must be maintained, in order to facilitate fertilization [72,73]. In our study, spermatozoa membrane functionality deteriorated gradually with time of storage. Supplementation of the extender with cryoprotectants significantly enhanced spermatozoa membrane functionality of liquid-stored buck semen, at all intervals checked. No significant difference among the three commercial extenders was noted. These results agree with a study on ram semen liquid storage, that reports an equal protection of membrane integrity between a 2% soy lecithin extender and a standard egg yolk extender [64]. Our results are also in line with studies on buck semen cryopreservation, which found no difference in the post-thaw membrane integrity of spermatozoa, frozen in soy lecithin or egg yolk extenders [31,47]. However, studies on buffalo semen cryopreservation report that a greater percentage of spermatozoa maintain post-thaw membrane integrity when extended in egg yolk, in comparison to soy lecithin extenders [74,75]. Surprisingly, there is a study on buffalo sperm cryopreservation that contradicts these results [76], but these contradictions can be attributed to the concentrations of cryoprotectants used, or the fine differences among extenders. Furthermore, a study on bull semen cryopreservation revealed that egg yolk protects membrane integrity of bull spermatozoa more efficiently, in comparison to soy lecithin, during the equilibration period [62].

Spermatozoa viability is an essential parameter for fertilization [77]. Viability results of the present experiment followed the patterns of spermatozoa cell membrane integrity. Spermatozoa viability appeared to reduce significantly as storage time increased, while supplementation of the extender with cryoprotectants enhanced liquid-stored spermatozoa viability. Our results on spermatozoa viability are in line with buck semen cryopreservation studies [31,47]. Soy lecithin (OviXcell^®^) or egg yolk lecithin (Steridyl^®^) extenders offered more efficient protection of spermatozoa viability than the extender based on plant phospholipids (AndroMed^®^), which only tended to differ from the basic extender. These differences can be attributed to the abovementioned differences in the constitution of the two plant phospholipid-based extenders.

Interestingly, OviXcell^®^ (SL) offers a better protection to spermatozoa viability, while AndroMed^®^ (PP) preserves spermatozoa motility in a more efficient way. This result may be linked to differences in the form, function and origin of the plant phospholipids that are contained in the extenders [56], or else it can depend on differences in the other ingredients of the extenders (concentration of glycerol, combination of buffers, choice of sugars).

Spermatozoa acrosome integrity declined significantly over time of storage, in all extenders used. All commercial extenders containing cryoprotectants, were more efficient in protecting acrosome integrity, in comparison to the basic simple extender. The egg yolk-based extender tended to offer superior protection to the acrosome membrane, in comparison to the two plant phospholipid-based extenders, over the 48 h storage period. Interestingly, in contrast to our results, a recent buck semen cryopreservation study has shown that egg yolk- and soy lecithin-based extenders did not differ in the efficacy of post-thaw spermatozoa acrosome membrane protection, while they also did not differ in kidding rates in a fertility trial [47]. However, there is a study in buffalo semen cryopreservation which reveals an exciting detail; although post-thaw acrosome integrity did not differ, between the egg yolk- and the soy lecithin-based extenders, the egg yolk-based extender yielded significantly superior results, both in the in vitro inducibility of acrosomal exocytosis and in the fertility trial [75]. These results are in line with a study on bull semen cryopreservation, which indicates that egg yolk extenders are more efficient in maintaining post-thaw acrosome integrity of bull spermatozoa [50].

The results of the present study clearly indicate the superiority of the egg yolk- and plant phospholipid-based extenders, supplemented with glycerol, in comparison to the basic extender. Taking into account that all the commercial extenders contain glycerol, while the basic extender does not, our results could be also influenced by the presence of glycerol and its concentration in the extenders. Glycerol is capable of permeating the cell membrane of spermatozoa, but it also prompts changes in an extra- and intracellular level [78]. Glycerol is only non-toxic to cells if the appropriate concentration is used, while buck spermatozoa freezability is favored by glycerol addition, at a concentration of 5–9%, which ranges depending on the goat breed [79].

Furthermore, the slight superiority of the egg yolk extender in preserving spermatozoa motility and acrosome integrity, over time of storage, does not readily suggest that the egg yolk extender might yield improved fertility results in a future fertility trial. Chelucci et al. [70] reported that the egg yolk extender was superior to the soy lecithin extender in preserving frozen-thawed buck spermatozoa viability and motility, but this result was reversed when a heterologous in vitro fertilization test has shown higher fertilization rates for the soy lecithin extender. Studies on bull semen suggest that sperm quality characteristics appear enhanced in egg yolk extenders, in comparison to soy lecithin extenders [50,60,61,80], while there are studies confirming greater in vivo fertility, after preservation in egg yolk extenders, when compared to soy lecithin extenders [81,82]. However, Gil et al. [83] reported no differences in the fertility of cryopreserved ram semen between egg yolk and soy lecithin extenders, while others report superior protection of the soy-based, in comparison to the egg yolk extenders during cryopreservation [59,84]. Finally, in a bull semen cryopreservation study, significant differences among extenders, in post-thaw spermatozoa motility and kinetics, were not reflected on a fertility trial [85].

Apart from differences in storage protocols [86], the debate that concerns the comparison among extenders is also relevant to species differences, since spermatozoa and seminal plasma from different species react to cryoprotectants during storage in a different way [87]. Some Authors also report goat breed differences in spermatozoa sensitivity to the components of the extender [88,89,90,91], a fact that should also be taken into account.

Consequently, in vitro fertilization tests and fertility trials are necessary to validate the results of the present study on liquid Skopelos buck semen.

## 5. Conclusions

Plant phospholipid-based extenders seem to preserve sperm quality parameters in a pattern similar to the egg yolk-based extender, in liquid Skopelos buck semen. The extender based on soy lecithin (OviXcell^®^) was superior to the plant phospholipid-based extender (AndroMed^®^) in preserving viability, while the second was more effective than the first in protecting total and progressive spermatozoa motility. However, it seems that the extender based on egg yolk lecithin (Steridyl^®^) is still better in maintaining core sperm qualities, which are correlated to high fertility rates after artificial insemination, such as spermatozoa motility and acrosome integrity. Fertility trials in Skopelos goats are necessary to clarify these effects.

## Figures and Tables

**Table 1:** Percentage (mean ± SE) of CASA kinetic parameters measured in Skopelos buck semen diluted with extenders containing soy lecithin (OviΧcell^®^), plant phospholipids (AndroMed^®^), egg yolk lecithin (Steridyl^®^) or no phospholipids (basic extender) after 0, 24 and 48 h of liquid storage.

		1—Soy Lecithin	2—Plant Phospholipids	3—Egg Yolk Lecithin	4—Basic Extender
VCL	0 h	63.69 ± 5.74	71.76 ± 5.74	103.66 ± 6.63	63.91 ± 5.74
	24 h	56.61 ± 5.99	63.74 ± 5.74	67.64 ± 6.29	64.88 ± 5.74
	48 h	42.86 ± 6.29	50.99 ± 6.29	68.47 ± 7.51	52.93 ± 6.29
VSL	0 h	39.67 ± 4.83	40.10 ± 4.83	70.48 ± 5.57	41.35 ± 4.83
	24 h	32.62 ± 5.04	29.62 ± 4.83	42.14 ± 5.29	40.68 ± 4.83
	48 h	20.61 ± 5.29	21.00 ± 5.29	43.91 ± 6.32	32.11 ± 5.29
VAP	0 h	46.50 ± 5.37	50.09 ± 5.37	88.21 ± 6.20	52.21 ± 5.37
	24 h	39.89 ± 5.61	37.50 ± 5.37	54.56 ± 5.88	51.27 ± 5.37
	48 h	26.82 ± 5.88	28.66 ± 5.88	55.06 ± 7.03	40.05 ± 5.88
LIN	0 h	55.44 ± 3.79	48.93 ± 3.79	60.80 ± 4.37	55.38 ± 3.79
	24 h	46.40 ± 3.95	40.44 ± 3.79	52.48 ± 4.15	52.37 ± 3.79
	48 h	39.74 ± 4.15	34.48 ± 4.15	53.16 ± 4.96	54.50 ± 4.15
STR	0 h	70.14 ± 3.10	70.97 ± 3.10	72.75 ± 3.58	70.26 ± 3.10
	24 h	69.26 ± 3.23	69.70 ± 3.10	67.33 ± 3.39	70.34 ± 3.10
	48 h	65.69 ± 3.39	61.85 ± 3.39	67.68 ± 4.06	70.29 ± 3.39
WOB	0 h	67.89 ± 3.07	65.00 ± 3.07	79.75 ± 3.54	73.59 ± 3.07
	24 h	62.48 ± 3.20	55.38 ± 3.07	72.78 ± 3.36	73.44 ± 3.07
	48 h	57.53 ± 3.36	52.77 ± 3.36	72.96 ± 4.02	70.05 ± 3.36
ALH	0 h	2.22 ± 0.13	2.66 ± 0.13	2.69 ± 0.15	2.07 ± 0.13
	24 h	2.12 ± 0.13	2.64 ± 0.13	2.23 ± 0.14	2.19 ± 0.13
	48 h	1.89 ± 0.14	2.38 ± 0.14	2.18 ± 0.17	1.97 ± 0.14
BCF	0 h	7.28 ± 0.69	8.27 ± 0.69	7.19 ± 0.80	6.03 ± 0.69
	24 h	6.78 ± 0.73	8.91 ± 0.69	5.11 ± 0.76	6.12 ± 0.69
	48 h	5.56 ± 0.76	6.86 ± 0.76	5.36 ± 0.91	5.55 ± 0.76

VCL (curvilinear velocity, µm/s); VSL (straight-line velocity, µm/s); VAP (average path velocity, µm/s); LIN (VSL/VCL, %); STR (VSL/VAP, %); WOB (VAP/VCL, %); ALH (amplitude of lateral head displacement, μm); BCF (beat cross frequency, Hz). [VCL, VSL, VAP: Extender: 3 > 1 = 2 = 4, *p* < 0.00005; Time (h): 0 > 24 > 48, *p* < 0.00005. LIN, WOB: Extender: 3 = 4 > 1 = 2, *p* < 0.00005; Time (h): 0 > 24 = 48, *p* < 0.005. ALH: Extender: 2 = 3 > 1 = 4, *p* < 0.00005; Time (h): 0 = 24 > 48, *p* < 0.005. BCF: Extender: 2 > 1 = 3 = 4; *p* < 0.05; Time (h): 0 = 24 > 48, *p* < 0.005. STR: Extender: 3 = 1 = 2 = 4, *p* > 0.05; Time (h): 0 = 24 = 48, *p* > 0.05 (*p* is provided for comparisons between extenders within time and between time within extenders)].

## Data Availability

Data supporting the reported results are available to anyone interested after justified application is provided.
